# Dynamic Mechanical Analysis and Ballistic Performance of Kenaf Fiber-Reinforced Epoxy Composites

**DOI:** 10.3390/polym14173629

**Published:** 2022-09-02

**Authors:** Thuane Teixeira da Silva, Pedro Henrique Poubel Mendonça da Silveira, André Ben-Hur da Silva Figueiredo, Sérgio Neves Monteiro, Matheus Pereira Ribeiro, Lucas de Mendonça Neuba, Noan Tonini Simonassi, Fabio da Costa Garcia Filho, Lucio Fabio Cassiano Nascimento

**Affiliations:** 1 Department of Materials Science, Military Institute of Engineering—IME, Praça General Tibúrcio 80, Urca, Rio de Janeiro 22290-270, Brazil; 2 Advanced Materials Laboratory (LAMAV), Department of Materials Engineering, State University of the Northern Rio de Janeiro—UENF, Avenida Alberto Lamego, 2000, Campos dos Goytacazes 28013-602, Brazil

**Keywords:** kenaf fiber, dynamic-mechanical analysis (DMA), dynamic mechanical, epoxy composite, ballistic test

## Abstract

Several industry sectors have sought to develop materials that combine lightness, strength and cost-effectiveness. Natural lignocellulosic natural fibers have demonstrated to be efficient in replacing synthetic fibers, owing to several advantages such as costs 50% lower than that of synthetic fibers and promising mechanical specific properties. Polymeric matrix composites that use kenaf fibers as reinforcement have shown strength increases of over 600%. This work aims to evaluate the performance of epoxy matrix composites reinforced with kenaf fibers, by means of dynamic-mechanical analysis (DMA) and ballistic test. Through DMA, it was possible to obtain the curves of storage modulus (E′), loss modulus (E″) and damping factor, Tan δ, of the composites. The variation of E′ displayed an increase from 1540 MPa for the plain epoxy to 6550 MPa for the 30 vol.% kenaf fiber composites, which evidences the increase in viscoelastic stiffness of the composite. The increase in kenaf fiber content induced greater internal friction, resulting in superior E″. The Tan δ was considerably reduced with increasing reinforcement fraction, indicating better interfacial adhesion between the fiber and the matrix. Ballistic tests against 0.22 caliber ammunition revealed similar performance in terms of both residual and limit velocities for plain epoxy and 30 vol.% kenaf fiber composites. These results confirm the use of kenaf fiber as a promising reinforcement of polymer composites for automotive parts and encourage its possible application as a ballistic armor component.

## 1. Introduction

Several industrial sectors, e.g., automotive and aeronautics, seek to develop lighter and more resistant materials as a way of increasing mechanical performance [1,2]. The composite materials, mainly those comprising a polymeric matrix reinforced with fibers, fit well in answering to these demands owing to their high stiffness capacity and strength improvement associated with lower density [3], as well as favorable cost-effectiveness. As a continuous demand effect, some alternatives to the broadly synthetic fibers, specially glass fibers, have recently been increasingly explored in view of even cheaper materials with few differences in properties [4,5]. Indeed, an increase in research works related to natural lignocellulosic fibers (NLFs) as reinforcement in polymeric matrices is currently observed. NLFs are already able to promote characteristics similar to composites with lower density and cost [6].

Today, most investigated and industrially used NLFs, such as jute [7,8,9], sisal [10,11,12], hemp [13,14,15,16], flax [17,18,19], and cotton [20,21,22], are considered in several applications owing to their eco-friendly character associated with lower processing energy [23]. However, the renewability of NLFs depends on the polymer matrix as well as superficial treatments used to increase the adhesion between fiber and matrix [24].

Composites with a polymer matrix reinforced with NLFs bear considerable potential for application in automotive, furniture, packing, and armor industries [25,26,27,28]. Although those reinforced with synthetic fibers present higher mechanical performance, these composites also bear a considerable negative impact on the ecosystem, due primarily to the use of petroleum as raw material as highlighted by recent studies [29,30]. Therefore, NLFs provide a more eco-friendly character to the final material and minimize the global dependency on fossil fuels, in addition to favoring a broader range of manufacturers [31]. On the other hand, some relevant remarks must be taken into account before considering its application, such as NLF hydrophilic character, poor adhesion to the polymeric matrix [32]; non-uniform properties due to intrinsic factors such as age, extraction process, and environmental conditions [33]; low thermal stability [34]; as well as low mechanic performance compared to the synthetic fibers [35].

**Figure 1:**
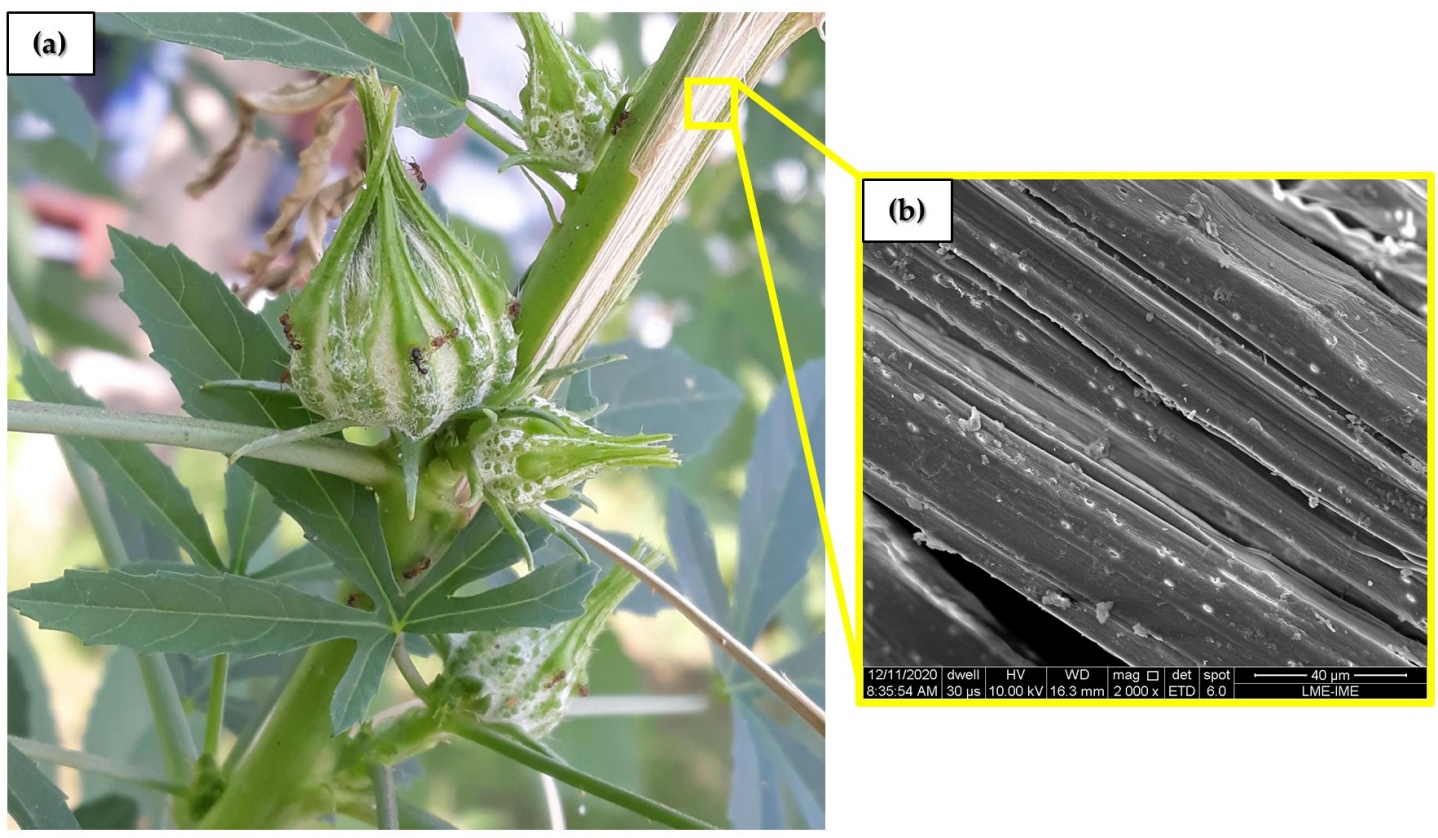
(**a**) Stem of the kenaf plant; (**b**) SEM of the kenaf fiber longitudinal surface.

Kenaf fiber (*Hibiscus cannabinus* L.) is one of the most commonly used NLFs as reinforcement in polymer matrix composites and in several other industrial applications [36]. In 2020, the total harvest of kenaf crops globally yielded 126,000 tons [37]. These fibers have a significant advantage in terms of growth rate; about 3 meters in 3 months and at roughly 5 cm of thickness, even in adverse weather conditions [38]. The kenaf plant, illustrated in Figure 1a, provides distinct fibers extracted from different parts (leaves, stalks, seeds) [39], as long as sufficient care is taken during planting, growing, and harvesting [40,41]. Figure 1b illustrates the kenaf fiber surface under high magnification.

In recent works, promising results are observed for kenaf fibers as reinforcement in different types of polymer matrices, such as HDPE [42,43,44,45], PP [46,47,48,49], polyester [50,51,52], epoxy [32,53,54,55], polylactic acid (PLA) [56,57,58,59], polystyrene [60,61], and PVC [62,63]. In particular, Ochi [64] reported an increase of 687% in tensile strength of 70 wt% kenaf fiber composites as compared to plain PLA. Many works have already highlighted the dynamic-mechanic properties of kenaf fibers [58,63,65,66,67]. Datta and Kopczynska [65] studied the dynamic properties, absorption and morphology of kenaf fibers treated with acetylation in a polyurethane matrix. The composites showed high damping capacity, with Tan δ less than 0.2. Woo and Cho [58] analyzed the effects of ammonium polyphosphate in the thermal and mechanical properties of a kenaf/PLA composite through dynamic-mechanical analysis (DMA). The storage module exhibited an increase of 165% in relation to unreinforced composites and the thermal and thermo-dimensional stabilities of the biocomposites were considerably increased. Bakar et al. [63] investigated the thermal properties of a kenaf-PVC/PVA composite. With the addition of kenaf fiber, the DMA curves indicated an increase in the glass transaction temperature of the composites. Saba et al. [68] studied the dynamic-mechanical properties of kenaf-epoxy filled with nano oil palm. The general results indicated that E′, E″ and T_g_ increased considerably with the incorporation of nanofibers from empty palm fruit bunches. Chee et al. [66] characterized the dynamic-mechanical properties of kenaf/bamboo–epoxy composites, the storage modulus of the hybrid composites before and after the glass transition region showed improvement following the addition of nanoclay. Azammi et al. [67] treated with alkalinization the kenaf fibers and used them as reinforcement in natural rubber (NR) and thermoplastic polyurethane to investigate its physical, viscoelastic and dynamic-mechanical properties. In the DMA test, an increase in damping properties at high temperatures (up to 135 °C) was observed. To our knowledge, kenaf-fiber-reinforced epoxy composites have not yet been investigated for both DMA and ballistic performance. Thus, the objective of this work is to investigate for the first time, the influence of the addition of different amounts of kenaf fiber, up to 30 vol.%, on the DMA properties of epoxy composites. From the aforementioned information, it can be noted that no investigation have so far been carried out on the properties obtained from the DMA of kenaf/epoxy composites, and their ballistic performance. The worldwide cultivated and industrially applied kenaf fiber together with its superior mechanical performance as reinforcement in polymer composites were the appealing motivations to perform this research.

## 2. Materials and Methods

### 2.1. Materials

The kenaf fibers, illustrated in Figure 1, extracted from the *Hibiscus cannabinus* stalks, were supplied by the Tapete São Carlos, from São Paulo, Brazil. The as-received kenaf fibers were manually cleaned and then dried at 60 °C for 24 h, as commonly used for NLFs [69]. The fibers were not subjected to any chemical treatment. The epoxy resin used was a diglycidyl ether bisphenol A (DGEBA), hardened with triethylenetetramine (TETA) in a stoichiometric ratio of 100:13, as recommended by the manufacturer, Merck. Both resin components were supplied by Resinpoxy Ltda., Rio de Janeiro, Brazil.

### 2.2. Fabrication of Composites

**Figure 2:**
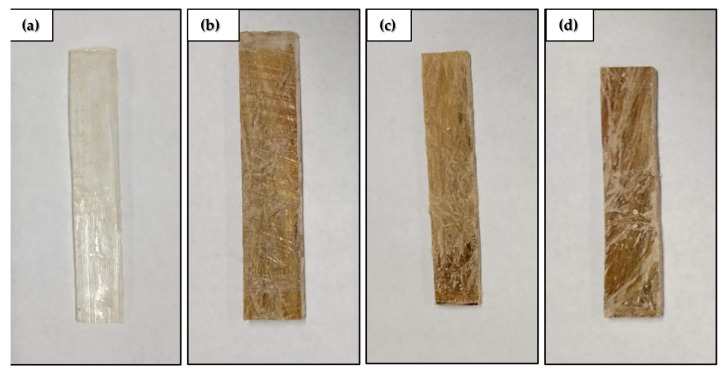
Samples for DMA testing: (**a**) epoxy resin; (**b**) 10 vol%, (**c**) 20 vol% fibers and (**d**) 30 vol% fibers.

Composite plates were produced by a compression process in a 150 × 120 mm metallic mold, 10 mm in thickness, based on ASTM D4065-01 [70], and 10, 20, and 30 vol% kenaf fibers, applying a pressure of 5 MPa for 24 h, as commonly used for NLF-based epoxy composites [16,26,71]. Aligned kenaf fibers were carefully laid inside the mold with a still fluid resin-hardener mix at a predefined proportion of fiber and resin. The epoxy resin density was considered equal to 1.11 g/cm³, the same as that found in the literature [71,72], and the fiber density was evaluated through tests, obtaining an average of 1.52 g/cm³ [71]. Figure 2 shows the specimens that were prepared for testing, according to ASTM D4065-01 [70].

### 2.3. Dynamic Mechanical Analysis

The DMA test was performed according to ASTM D4065-01 [70] to identify the parameters of storage modulus (E′), loss modulus (E″), and tangent delta (Tan δ) obtained in the test. The equipment model Q800 (TA Instruments, New Castle, DE, USA) was used, operating at a frequency of 1 Hz, in temperatures ranging from 30 to 200 °C, with a heating rate of 3 °C/min, under a nitrogen atmosphere. The samples were subjected to the three-point bend test, where the dimensions 64 × 13 × 3 mm were used in the samples. Table 1 describes the nomenclature of the samples according to the fiber content adopted in the composite.

### 2.4. Ballistic Tests

Ballistics tests were carried out in a related facility at the Military Institute of Engineering, Rio de Janeiro, Brazil. A gunpower SSS pressure rifle (Gunpower, UK) using 0.22 caliber lead ammunition with nominal 3.3 g of mass, was positioned 5 m away from the target face aligned 90 degrees (perpendicular) to the projectile trajectory, as recommended by standard NIJ 0101.06 [73]. Both impact ($V_{i}$) and residual ($V_{r}$) velocities were measured by means of two Air Chrony model MK3 ballistic chronographs, one positioned 10 cm in front and the other 10 cm behind the target, respectively. The energy absorbed by the target ($E_{abs}$) was calculated as [74]:

(1) $$E_{abs}=\frac{m(V_{i}^{2}-V_{r}^{2})}{2}-E_{abs}$$

where *m* is the projectile mass and $E_{abs}$ the absorbed energy during the projectile flight without target. Based on the calculated value of $E_{abs}$ in Equation (1), an important ballistic parameter, namely the limit velocity ($V_{L}$) can be evaluated by Equation (2) [74]:

(2) $$V_{L}=\sqrt{\frac{2\cdot E_{abs}}{m}}$$

### 2.5. Scanning Electron Microscopy (SEM)

The kenaf fiber surface, as well as the kenaf–epoxy interface, were analyzed using a Quanta FEG 250 microscope, Fei (Hills-Boron, Hillsboro, OR, USA) with a secondary electron detector, an accelerating voltage of 10 kV, and a magnification ranging from 240 to 1600×. The fibers were covered with gold in the Leica Ace600 equipment (Wetzlar, Germany).

## 3. Results

### 3.1. Storage Modulus (E′)

The storage modulus represents the elastic behavior of a material when subjected to sinusoidal stress. The storage modulus provides information about the dynamic-mechanical properties of a material, such as stiffness, load capacity, crosslink density, and interfacial strength between fiber and matrix [69,75,76,77]. A clear understanding of the storage module provides important information about the stiffness, degree of crosslinking, and fiber/matrix interfacial bonding.

**Figure 3:**
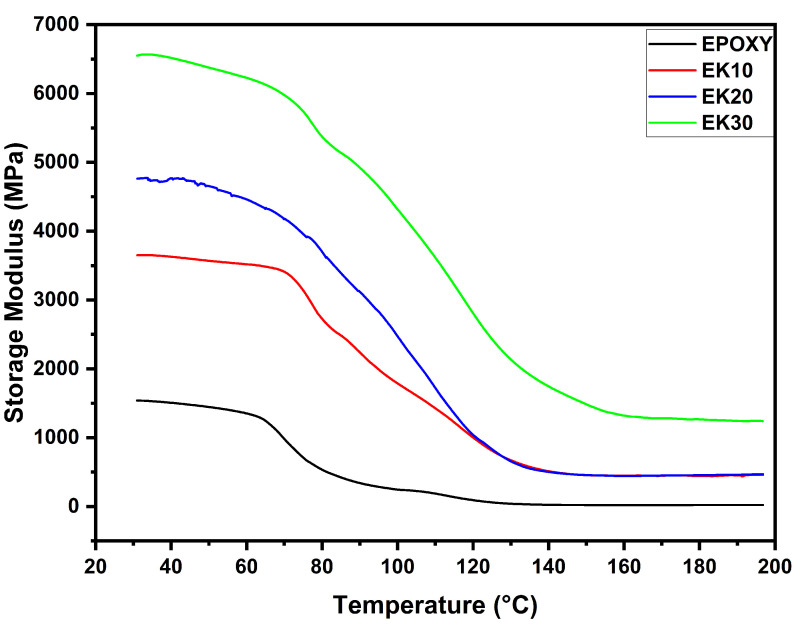
DMA storage modulus (E′) curves for epoxy composites incorporated with different amounts of kenaf fibers.

Figure 3 illustrates the E′ curves variation with temperature for the kenaf/epoxy composites. It can be noted that the increase in temperature caused a drop in the storage modulus in all compositions. The E′ results obtained are similar to those presented by Oliveira et al. [69], which produced epoxy matrix composites reinforced with thermally aged fique fabric. The curves of E′ become wider in the glassy region, located between 50 and 150 °C. The increase in kenaf fiber volume fiber caused an increase in the storage modulus of all composites. The drop in curve E′ starts at approximately 63 °C for EPOXY, EK20 and EK30 samples, and at 69 °C for EK10, with the drop in the curve ending in the region between 140 and 160 °C for composites EK10, EK20 and EK30, and at 120 °C for EPOXY.

The region where the E′ drop occurs is defined as the glass transition region (T_g_) of the composite, indicating the movement of the main polymeric chain [78]. Below the glass transition region, the polymeric chain movement is restricted due to the low mobility of the frozen and packed molecule arrangement. Thus, E′ has a high value in the glassy state. With the increase in temperature, the arrangement of packed molecules collapses, causing the polymer chain to acquire high molecular mobility and increasing free volume components, resulting in the storage modulus dropping and moving to the viscoelastic region of the material. Table 2 shows the values of the storage module of the composites.

### 3.2. Loss Modulus (E″)

**Figure 4:**
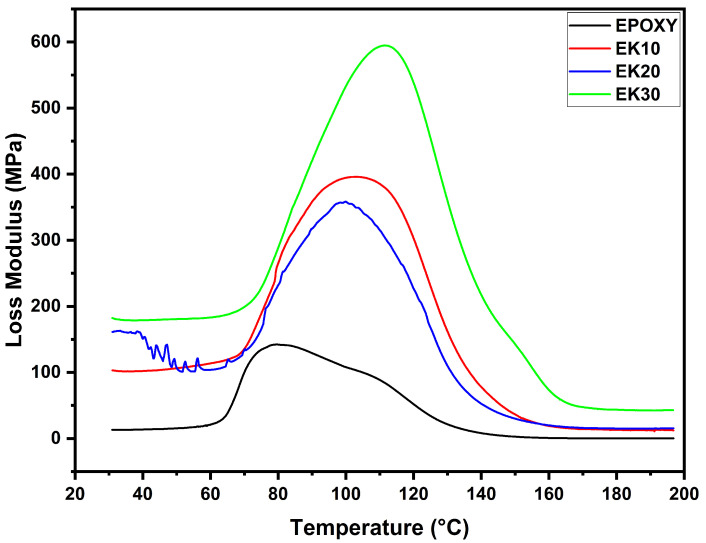
DMA loss modulus (E″) curves for epoxy composites incorporated with different amounts of kenaf fibers.

The loss modulus (E″) shows the viscous behavior of a material when subjected to an oscillating stress cycle [78]. A material with a high E″ value indicates that it has a higher energy dissipation capacity and therefore better damping properties to reduce the damaging forces caused by mechanical energy. Figure 4 shows the E″ vs. temperature curves for neat epoxy and epoxy/kenaf composites.

All composites reached a maximum peak height in the glass transition region; the values are presented in Table 2. As shown in Figure 4, the glass transition region starts at 70 °C. In this region, the loss modulus is low and constant. However, in the rubbery state the viscous behavior of the material increases. This occurs via superposition of the molecular segment motion in the polymeric chain with mechanical deformation, resulting in high internal friction and inelastic deformation [79]. This results in a high dissipation energy, with the loss modulus reaching the highest point of the peak, related to T_g_ of the material. After reaching this point, the polymeric molecules pass to a relaxed state and reduce their internal friction. This reduction in friction causes a drop in the loss modulus. Neat epoxy has the lowest E″ value (0.14 GPa), but with the addition of kenaf fibers an increase is observed in the loss modulus of the composites of about 178.5% for EK10, 150% for EK20, and 321.4% for EK30. The increase in kenaf fiber content as reinforcement induced greater internal friction, resulting in a higher E′’ value.

### 3.3. Damping Factor (Tan δ)

**Figure 5:**
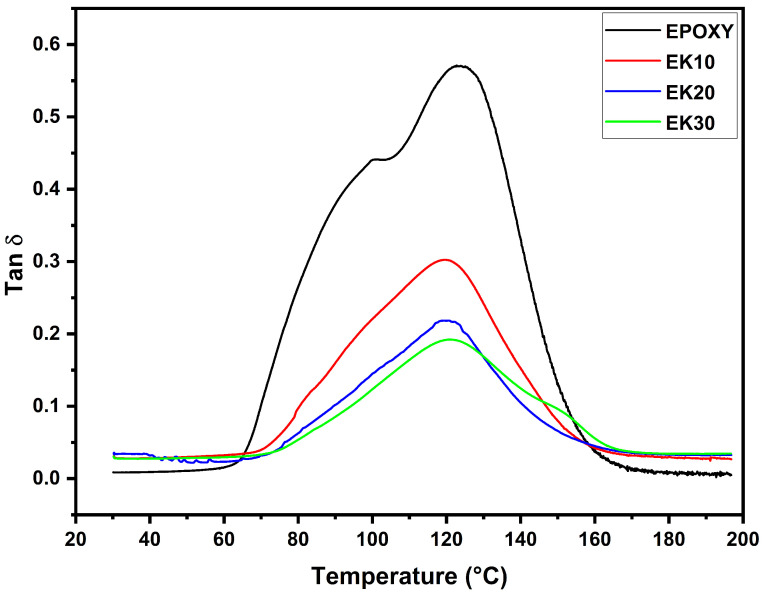
Damping factor (Tanδ) curves for epoxy composites incorporated with different amounts of kenaf fibers.

The Tan δ curve, also called loss ratio or damping factor, is obtained from the ratio between the loss modulus and storage modulus (E″/E′) and is associated with the heat dissipation during each deformation cycle and an elastic behavior of the material. Thus, from Figure 5, the consequence of kenaf addition and the temperature variation on the composites damping properties can be noted. The Tan δ values of epoxy–kenaf (EK) composites (0.19–0.30) in Figure 5 are, in every case, lower than EPOXY (0.57). These results indicate that the neat epoxy presents a higher damping factor, which is related to a greater inelastic deformation with a higher energy dissipation. On the other hand, the decrease of the peak of the EK groups can be attributed to the interlocking mechanism between the fibers and the polymer matrix, which restricts the chain movement [80]. This is visualized where the EK30 presented the lowest Tan δ peak height (0.19) in Figure 5 among the three other groups, indicating strong interfacial interaction and lower energy dissipation at the interface [81,82].

### 3.4. Cole–Cole Plot

**Figure 6:**
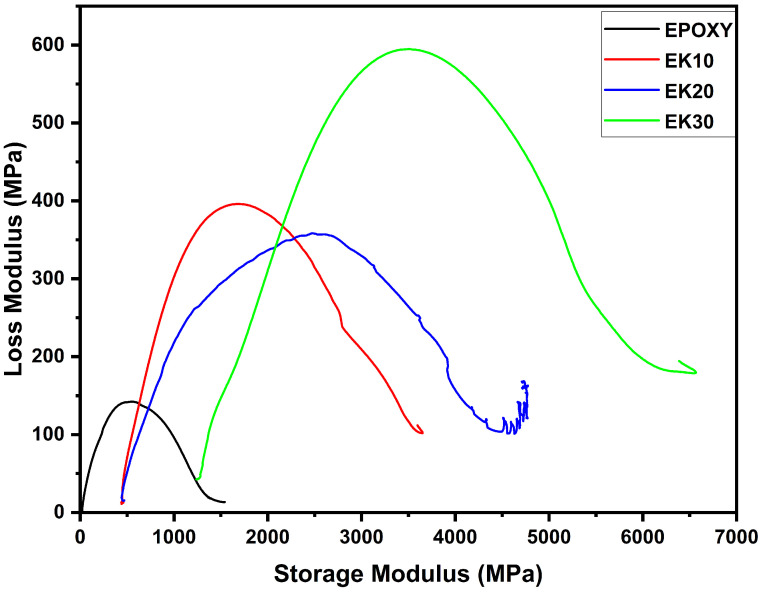
Cole–Cole plot for epoxy composites incorporated with different amounts of kenaf fibers.

**Figure 7:**
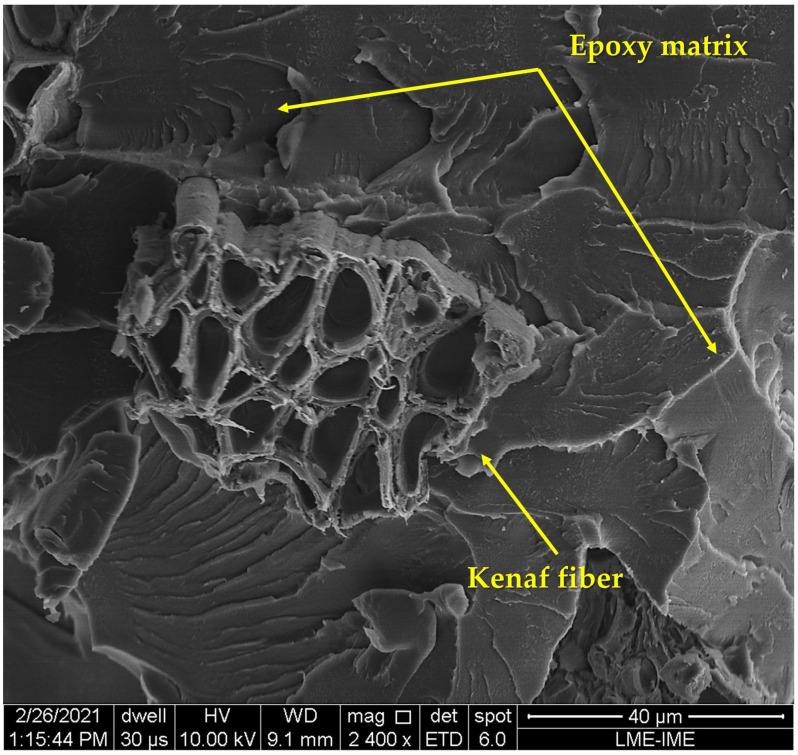
SEM micrograph illustrating a kenaf fiber in the epoxy matrix.

The Cole–Cole graph is obtained from the relationship between the loss modulus (E″) and the storage modulus (E′) [83], from which semicircular curves for the homogeneous polymeric system [84] are obtained and related to effects present at the interface and heterogeneous dispersion of phases [85]. Thus, Figure 6 shows a regular curve for neat epoxy and a more irregular curve for EK composites, which increase for larger volumes of natural reinforcement. As aforementioned, these curves indicate a greater heterogeneous dispersion for these materials due to the presence of kenaf fibers in the polymeric structures of the resin, Figure 7 which represents a greater interfacial interaction between kenaf and epoxy in these fractions.

The results obtained are similar to the findings from previous research, in which the palm–epoxy combination in fractions of 40, 50, and 60 vol%, presented semicircular curves with greater irregularities as the reinforcement volume increased [85]. Similar behavior was also observed in the work reported by Vijayan et al. [86] for the Aloe Vera–epoxy composite.

### 3.5. Ballistic Test Results

The ballistic tests of ten 0.22 projectiles shot against 10 mm thick target plates with 150 × 120 mm of epoxy surface for both plain epoxy and 30 vol% kenaf fiber composites, resulted in corresponding impact ($V_{i}$) and residual ($V_{r}$) velocities as well as the parameters, calculated from Equations (1) and (2) and based on the NIJ 0101.06 standard [73], presented in Table 3.

**Figure 8:**
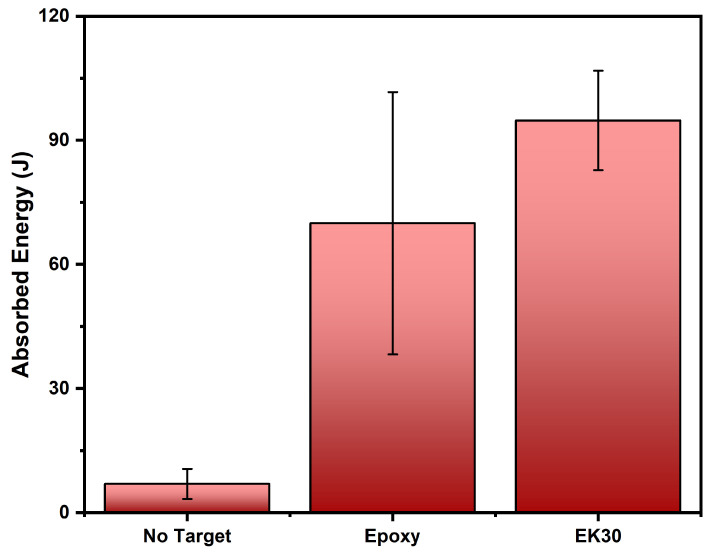
Values of ballistic absorbed energy against 0.22 caliber ammunition of plain epoxy, 30 vol% kenaf fiber composite and shooting condition without actual target.

To compare the ballistic absorbed energies, Figure 8 shows the values of $E_{abs}$ from Table 3 including the corresponding error bars. Despite the greater mean value of absorber energy, for the composite, the relatively higher values of the standard deviations render them similar. The V_L_ of EK30 in Table 3 is superior to that reported for epoxy composites reinforced with the same 30 vol% amount of caranan and tucum fibers [87,88] as well as sedge fiber [89]. An ongoing investigation with an increased number of shootings is expected to statistically confirm the superior ballistic performance of kenaf-fiber-reinforced epoxy composites.

### 3.6. Microstructure

The morphological observation of the tested samples allowed one to observe the interaction between fiber and matrix, representing a fundamental aspect regarding the mechanical properties of the material.

**Figure 9:**
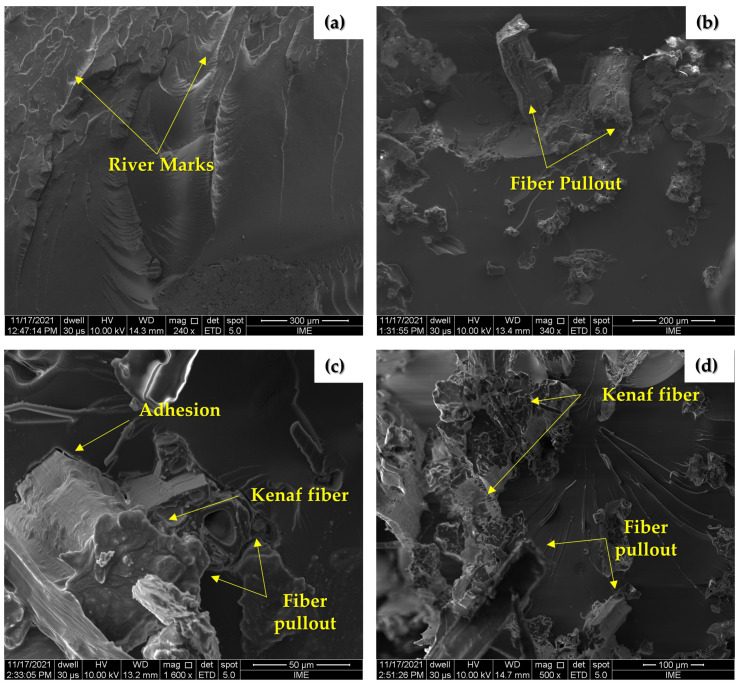
SEM images of fractured samples: (**a**) EPOXY; (**b**) EK10; (**c**) EK20; (**d**) EK30.

In Figure 9, one can observe in the fractography of the neat epoxy (Figure 9a) the high density of river marks, which indicates a brittle fracture due to the absence of reinforcement. In the micrograph of EK10 (Figure 9b), one can observed a few pulled-out fibers, which can be attributed to the low interfacial adhesion between the fiber and the polymeric matrix due to the presence of voids and cracks. As for the EK20 and EK30 composites (Figure 9c,d), they have an even greater pullout density due to an increase in the adhesion of the reinforcement to the matrix. This can be related to relevant mechanical properties with respect to the other groups. Indeed, recent ballistic results for epoxy composites reinforced with caranan fiber [87] displayed cracks and delamination. This indicates an adhesion to the matrix less efficient than that of kenaf fiber revealed in Figure 7 and Figure 9.

## 4. Summary and Conclusions

Kenaf/epoxy composites were produced in three different volumes of fibers, 0, 10, 20 and 30 vol%. From the results obtained via DMA and SEM images, the following conclusions can be made:

- The storage modulus (E′) of every composite presented a considerable decrease at 60 °C, around the glass transition temperature, where a movement in the polymeric chains occurs and provides a rubbery state to the material.
- The loss modulus (E′’) of the composites increased substantially as the volume fraction of kenaf also increased.
- The peaks presented on the damping factor (Tanδ) graph decreased with the volume fraction of kenaf increment in the composite. This could be related to an increase of the adhesion between fiber and matrix.
- In the Cole–Cole plot of the composites, a variation of the curves can be noted, specially for the neat epoxy, which appears close to a perfect semicircle. This could be associated with the lack of a second phase in the resin. The curves of the EK10, EK20, and EK30 composites presented an irregular semicircle, attributed to a greater heterogeneity of the material.
- Ballistic tests using 0.22 ammunition revealed similar performance in terms of absorbed energy and limit velocity for the plain epoxy and EK30 composite as targets. Despite the greater mean values for the EK30 composite, the relatively higher standard deviation remains inadequate in confirming the superior ballistic performance of the composite. A larger number of ballistic tests are required for the statistically supported determination of a definite conclusion.
- The fractographies of the composites after the tests showed crack regions where the stresses propagated through the composites. In addition, an increase in the adhesion across the kenaf/epoxy interface could be observed.
- The DMA and ballistic results, mainly regarding the 30 vol% kenaf-fiber-reinforced epoxy composite, confirmed not only the promising industrial applications in fields including automotive, civil construction, and furniture and packing, but also provide support for its application as a ballistic armor component.

## Figures and Tables

**Table 1:** Nomenclature of the composites used in this study.

Nomenclature	Composition
EPOXY	Neat Epoxy
EK10	10 vol.% Kenaf fiber
EK20	20 vol.% Kenaf fiber
EK30	30 vol.% Kenaf fiber

**Table 2:** Results of DMA analysis of the composites.

Composite	E′ at 30 °C (MPa)	E′ at 150 °C (MPa)	Peak of E″ (MPa)	Peak high of Tan δ
EPOXY	1540	20	140	0.57
EK10	4760	450	390	0.30
EK20	3640	450	350	0.22
EK30	6550	1480	590	0.19

**Table 3:** Ballistic results of ten shots using 0.22 projectile against plain epoxy (EPOXY) and 30 vol% kenaf fiber (EK30) composite.

Target	Projectile Mass (g)	$V_{i}$ (m/s)	$V_{r}$ (m/s)	$E_{\mathrm{abs}}$ (J)	$V_{L}$ (m/s)
No Target	3.11 ± 0.02	279.01 ± 13.10	275.98 ± 13.42	6.96 ± 3.62	-
Epoxy	3.10 ± 0.03	258.58 ± 36.20	158.56 ± 51.89	69.98 ± 31.69	212.48 ± 142.97
EK30	3.17 ± 0.01	288.30 ± 2.49	151.11 ± 12.01	94.81 ± 12.01	244.57 ± 87.05
